# Blue Notes: Using Songwriting to Improve Student Mental Health and Wellbeing. A Pilot Randomised Controlled Trial

**DOI:** 10.3389/fpsyg.2019.00423

**Published:** 2019-03-05

**Authors:** Kate A. Gee, Vanessa Hawes, Nicholas Alexander Cox

**Affiliations:** ^1^Centre for Performance Science, Royal College of Music, London, United Kingdom; ^2^School of Music and Performing Arts, Canterbury Christ Church University, Canterbury, United Kingdom; ^3^Independent Researcher, London, United Kingdom

**Keywords:** pilot RCT, mental health, songwriting, wellbeing, student, depression, anxiety, social identity

## Abstract

Higher Education is a period of transition. Students try out identities, develop skills, and explore their shifting sense-of-self. Recent evidence suggests an increase in mental distress in this population, pressurising in-house support schemes. Therapeutic songwriting is a music therapy technique, which can reduce mental distress and improve social engagement in a range of clinical populations; yet it is also an accessible art form, possibly an ideal vehicle for supporting students in distress. This paper examines whether participation in a weekly songwriting program could make a suitable RCT to support wellbeing within the HE environment. We used a methodologically rigorous pre-registered parallel wait-list pilot RCT design. Trial registration: ISRCTN11180007. Participants self-identifying as stressed, anxious, or depressed, or with a pre-existing mental health condition, were randomly allocated to the experimental group (5 weeks, songwriting) (*n* = 6) or to the wait-list control group (5 weeks, no intervention) (*n* = 6). Measures were taken at baseline and at the start (Time 1) and end (Time 2) of the intervention. Measures included: depression and anxiety scales, social identification, loneliness, and friendship. Change scores were calculated and a Mann–Whitney U revealed that depression levels in songwriters (*Mdn* = -1.0) differed significantly from wait-list controls (*Mdn* = 8.5) at T2, *U* = 5.00, *z* = -2.085, *p* < 0.041, *r* = 2.66. Songwriters’ levels of social connection (*Mdn* = 2.50) also differed significantly from wait-list controls (*Mdn* = 3.00) at T2 *U* = 3.00, *z* = -2.441, *p* < 0.015, *r* = 0.524. There were no other significant differences between control and intervention groups. A therapeutic songwriting intervention may have individual and group level benefits for a student population, alongside possible institutional benefits in student retention. Effects may be seen within depression and social connection metrics, and future RCTs should consider expanding measures for self-efficacy, social isolation, and wellbeing. This type of program illustrates a space for cost-effective, group, face to face additions institutional mental health support provision as part of a package of support for students.

## Introduction

2006/7–2015/16 saw a 350% increase in the disclosure of mental health conditions by UK first year students in Higher Education (Thorley, 2017). The National Union of Students first acknowledged this concern in 2013 (Kerr, 2013), suggesting approximately 20% of students considered themselves to have a mental health difficulty. Widespread coverage of poor student mental health (Southgate, 2019) has shaped a public narrative of a developing crisis in Higher Education. Growth in mental health difficulties parallels more than a decade of significant governmental changes to UK Higher Education including increasing fees, and the marketisation of education – which often negatively affects student expectations, drastically changes the experience of learning, and alters the demographic make-up of the student body. The most recent report (Thorley, 2017) suggests universities must see mental health and wellbeing as a priority. It is essential that funding be increased to develop dedicated university services, however, minimal guidance is given on types of provision. This research explores how music – in particular, songwriting as a group music-making tool – has the potential to support student wellbeing.

Cultural shifts in Higher Education, triggered by government, are changing how students consume and engage with learning, so, therefore, the ways in which we understand and engage with students needs to adapt. The current HE population living through these changes would readily benefit from applied research into engagement and wellbeing, but applied research into student experience tends to be based in Education and focuses on outcomes relating to student success (Sneyers and De Witte, 2018). There is limited research concerning musical interventions and student populations. In Daykin et al.’s (2018) systematic review of music and singing in adults, 10 out of 61 studies documented some use of music interventions with student populations. Music listening interventions to reduce negative mood states were most common (e.g., Boothby and Robbins, 2011). Notable exceptions were Valentine and Evans (2001) exploration of exercise versus solo or group singing, and Shwu Ming (2002) who used music therapy with students with mental health difficulties to reduce anxiety.

The field of community music-making for health develops interventions for populations with a range of physical, mental, and social struggles. A decade of research shows that music in general, and group singing in particular, can improve physical and mental health as well as wellbeing (Clift et al., 2016). Singing provides some benefit for a range of populations including older adults (Coulton et al., 2015) and can alleviate physical aspects of various conditions such as Parkinson’s Disease (Stegemöller et al., 2017), Chronic Obstructive Pulmonary Disease (Mcnamara et al., 2017), and Dementia (van der Steen et al., 2017), or to help manage broader problems such as trauma (von Lob et al., 2010).

Despite the dominance of singing as a music-making activity with subjective wellbeing benefits in the literature, it is not the only option. Songwriting is a prevalent and user-friendly art form, often taught by professional musicians, but engaged in by amateurs and professionals alike (Baker, 2015; Cohen and Miller, 2017). Frequently seen as a space for personal growth and development, many engage in the activity privately as a creative pursuit, but for music therapists it has become a tool in the armoury of therapeutic practice. Baker and Wigram (2005) define therapeutic songwriting as, “the process of creating, notating and/or recording lyrics and music by the client(s) and therapist within a therapeutic relationship to address psychosocial, emotional, cognitive, and communication needs of the client” (16).

Similar to singing, there has been a small but noticeable growth in therapeutic songwriting research (Baker, 2015). As a therapeutic intervention or clinical practice technique, songwriting has produced mixed results in changing subjective wellbeing indicators within clinical populations. For example, Hanser et al. (2006) found no significant differences in quality of life or psychological distress between conditions, but significant immediate effects in relaxation, comfort, happiness and heart rate. O’Brien (2014) found higher ratings in quality of life and physical wellbeing in a therapeutic group compared to controls, but no significant differences on psychological or existential wellbeing). There were also mixed results for depression (Silverman, 2013) with no significant between-group differences, although songwriters had the lowest mean depression scores compared to controls. Others, however, have illustrated that depression, anxiety, and relationships improved compared to controls (Choi et al., 2008). Songwriting has also been shown to affect broader concepts such as coping skills for illness management (Silverman, 2011; Klein and Silverman, 2012) and self-concept (Baker, 2015). It has also improved wellbeing in non-clinical populations (Baker et al., 2017). Of particular relevance to this study is work exploring changes in self-concept and meaning-making.

Songwriting enables writers to explore, question, and challenge their sense of self, personal narratives, and ways of being (Baker and MacDonald, 2013). Aasgaard and Ærø (2017, p. 654) note, “a song is a musical work of art. … Works of art are particular culturally emergent or culturally produced entities that are embedded in various human contexts. The fundamental nature of meaning of “a song” lies not in the song object, but “[…] in action, in what people do.”

The meaningfulness of songwriting as a product and as process is explored in depth (Baker et al., 2016a,b). Research suggests that songwriting operates through a “mechanism of change” (Baker et al., 2016b; Silverman et al., 2016). Examining this process, researchers have begun to untangle how songwriting may affect wellbeing. Meaning-making occurs in the outcomes and process of songwriting; work can be held up both as an achievement, and listened to at a later date, supporting ongoing exploration beyond the boundaries of the exercise itself. The creation of art objects as the outcome of research can raise ethical questions about the vulnerability of the participants and the sharing of work, but the process of involvement in artistic practice can have a significant impact upon wellbeing (Reason and Rowe, 2017). For example, the act of “performing” a song is noted to be a huge achievement for children who have struggled with low self-confidence (Davies, 2005).

Group songwriting may be particularly instructive to explore in a student wellbeing context because of its capacity to both create and explore social connections and identity. Within psychology, the social identity approach suggests that membership of social groups have a powerful impact on individual psychology. Social identity theory suggests that groups shape individuals’ attitudes, beliefs, and behaviour when they are internalised as social identities (Tajfel, 1978). It is known that isolation prevails in people experiencing mental health difficulties (Cromby et al., 2013) and that social connections, which are based in shared group memberships and social identities, can improve individual health and wellbeing (Jetten et al., 2009). Joining new groups can improve physical and mental health, even in those with severe depression (Cruwys et al., 2014). Conversely, a loss of identity will negatively affect wellbeing, and a transition to university may encompass many points of loss (Ng et al., 2018). Being in a period of transition involving the loss and creation of identities takes time to adjust to, and may affect some students more than others (Iyer et al., 2009). The creative, identity-based exploration of songwriting in a group setting may be a powerful tool with which to explore this transition and help students with these particular issues.

In comparison to allopathic approaches, managing wellbeing through clinical songwriting is difficult. Given the complexities of wellbeing and of songwriting as a mechanism, Baker et al. (2016a) calls for outcome studies to develop in four areas: to further isolate the aspects of wellbeing that are most positively impacted by songwriting; to encourage work across an array of populations to explore which are most responsive; to examine what length and frequency of songwriting sessions may lead to the best outcomes; and to consider which therapeutic orientation (if practiced) is the most effective.

### Pilot Study Design

The intention of this pilot study is to explore the space for an effective, larger RCT and improve its chances of success. Dissemination of the process of why and how an intervention could be undertaken and whether it is worthwhile supports others to avoid similar mistakes (Eldridge et al., 2016a,b; Thabane et al., 2016). This pilot randomised controlled trial enables evidence to build upon Baker’s suggestions through evaluating the process, benefits, and drawbacks of participating in a group songwriting workshop. As suggested in the most recent MRC guidelines for complex interventions (Craig et al., 2019, p. 27), “understanding of process can provide useful insights into why an intervention achieves or fails to achieve the expected outcomes” and that “it is important to provide a description of the intervention, and often useful to report the process of developing and implementing the intervention, as well as the results of the evaluation.” (p.31), which this paper aims to embody.

Music therapy interventions are complex interventions, often containing several interacting components, requiring clinical expertise for delivery, able to address numerous outcomes, and are often tailored to the recipient (Craig et al., 2008 cited in Robb and Burns, 2017). This complexity contributes to challenges in evaluating the efficacy and appropriateness of an intervention, hence the need to conduct a pilot trial alongside designing the pilot with two component studies: a pilot RCT, and a qualitative interview study exploring the process of songwriting as an intervention. This combined mixed-methods approach to pilot work enables the understanding of how an intervention might work in practice, the efficacy of recruitment, and ways in which an intervention may be altered and developed (O’Cathain et al., 2014). Although RCTs are sometimes adopted within music therapy research, they are relatively uncommon (Robb and Burns, 2017). A mixed-methods approach allows the testing of the efficacy of this intervention, the practicalities of its use, but also (and especially in the case of arts interventions) the understanding of the experience of its use by the participants. The current study adopts the recommended CONSORT extension approach suggested for developing pilot trials (Eldridge et al., 2016a).

Presented in this paper is the first part of the project, a pilot study employing a double randomised consent design, which was open and parallel. The study was conducted in the United Kingdom at Canterbury Christ Church University Psychology and Music Departments between January 2017 and April 2017. This study sought to assess the feasibility of using songwriting as an intervention within the university environment and the appropriateness of the intervention for this population within a future larger trial.

## Materials and Methods

### Ethics, Trial Registration

Ethical approval was granted by Canterbury Christ Church University (CCCU) Research Office (Project ID: 16/SAS/319C). The study was registered as a clinical trial on ISRCTN.com (11180007). The songwriting protocol was developed based on previous research and is discussed in 2.6 (Darnley-Smith and Patey, 2006; Baker, 2015; Baker et al., 2016b; Aasgaard and Ærø, 2017). Students were offered £20 vouchers for participation in the project.

### Study Design

Students were randomly allocated to the treatment group (songwriting) or the no-treatment group (wait-list control) before they gave consent to participate in the trial. The no-treatment group completed baseline measures (T1) and identical measures 2 months later (T2). Those allocated to the songwriting treatment group were offered the intervention and asked if they wished to take part in the trial. They completed baseline (T1) and Time 2 measures (T2).

### Participants

Students self-recruited through advertisements within two departments at CCCU. Those interested in participating completed a screening process Hospital Anxiety and Depression Scale (HADS), a short background survey of their mental health, and submitted a consent form. Inclusion criteria were students aged 18 or over, self-identifying as, “stressed, anxious, or depressed” and who were in their first year of study or new to either department. Exclusion criteria included students currently undergoing psychiatric care.

### Procedure

**FIGURE 1:**
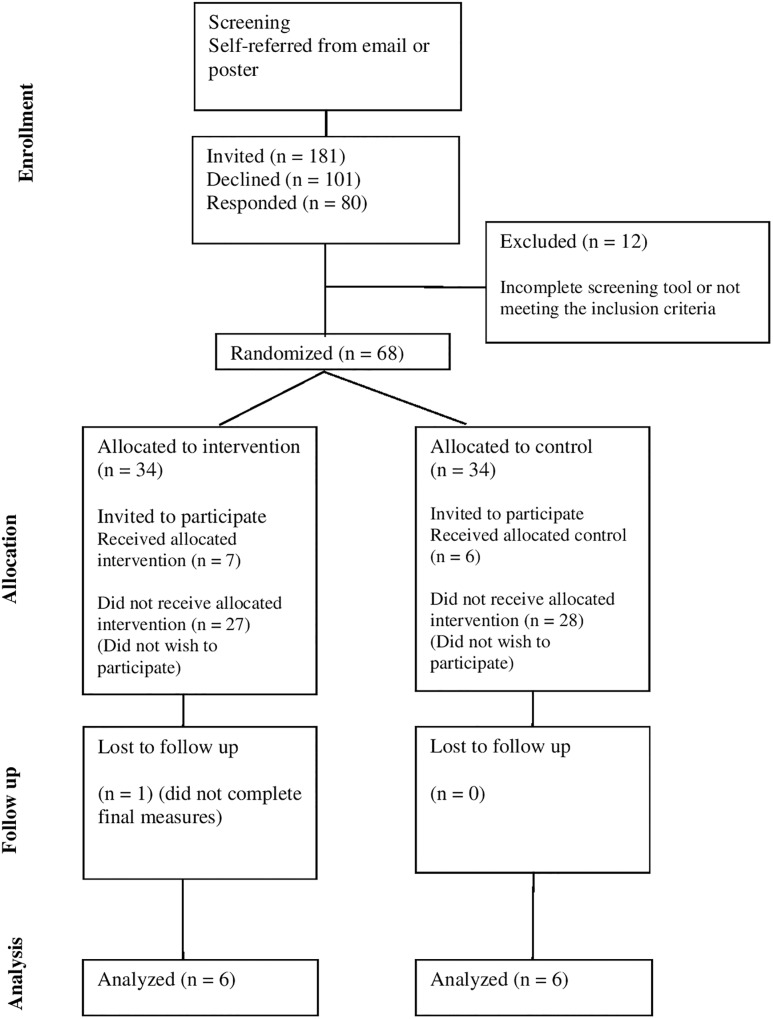
CONSORT diagram showing the flow of participants through each stage of the study.

Out of a potential pool of 181 (the number of first year students in two departments) 80 students registered their interest and completed an online screening questionnaire made up of sociodemographic and screening questions. All questionnaires were administered through a secure online survey platform^1^. Twelve students were excluded for an incomplete screening tool or not meeting the inclusion criteria. Sixty-eight students were randomised across both conditions. Randomisation was performed through a random number generator^2^. Thirty-four were randomly allocated to the treatment group and 34 to the no-treatment group. Students were then contacted, the study was explained in greater detail, and they were invited to take part. Seven students in the treatment group and six in the no-treatment group elected to take part in the study. One treatment group participant was lost to follow up when T2 measures were taken. A flow chart of the participants throughout the study is in Figure 1.

### Sociodemographics

All participants were female, with an age range of 18–31 years, and a mean age of 21.25 years. Fifty percent of the sample arrived at university with a pre-existing mental health condition; duration of condition varied: 10 years, 1–3 years, 3–5 years, and one participant being diagnosed within the previous 1–3 months. Others did not know or preferred not to say (see Table 1). Of those formally diagnosed with mental health conditions, all had co-morbid presentation. Conditions included: anxiety, depression, post-traumatic stress disorder, panic disorder, and personality disorder. The remainder had no formal diagnosis but self-identified as having depression, anxiety, or stress.

**Table 1:** Sociodemographic characteristics of participants pre-intervention.

Baseline characteristics	Songwriting intervention (*n* = 6)	Control (*n* = 6)	Full sample (*n* = 12)
**Gender:** *n* (% female)	6 (100)	6 (100)	12 (100)
**Age** (years): **M** *(SD)*	22.50 (4.593)	20 (2.0)	21.25 (3.621)
**Education level**			
1st year undergraduate: *n* (%)	5 (83.3)	6 (100)	11 (91.6)
1st year postgraduate: *n* (%)	1 (16.6)	0	1 (8.3)
**Current mental health condition: *n* (%)**			
Yes	3 (50)	3 (50)	6 (50)
No	0	1 (16.6)	1 (8.3)
Maybe	0	1 (16.6)	1 (8.3)
Don’t know	2 (33.3)	1 (16.6)	3 (25)
No but I have in the past	0	0	0
Prefer not to say	1 (16.6)	0	1 (8.3)
**Of those with a mental health condition (*n* = 6),**			
**Diagnosis included**			
Anxiety	2	3	(All had co-morbid diagnoses)
Depression	3	3	
Panic disorder	1	2	
Post-traumatic stress disorder	0	1	
Obsessive compulsive disorder	0	0	
Personality disorder	2	0	
**Of those with a mental health condition (*n* = 6),**			
**Condition presented for**: *n* (%)			
1–3 months	1	0	1 (16.6)
1–3 years	0	2	2 (33.3)
3–5 years	1	0	1 (16.6)
5–10 years	0	1	1 (16.6)
10+ years	1	0	1 (16.6)


### Songwriting Intervention

The study involved students creating and recording two songs over 5 weekly sessions. Songs were created in parallel, and by working with other participants, by using short creative exercises tapping into a framework introducing them to the lyrical and musical ideas involved in songwriting. Mental health symptoms were not the sole focus of the intervention. Exercises enabled students to explore their identities and wellbeing; they focused on exploring the sense of self, sense of place, friendships, aspirations, and emotions relating to university. Students were asked to focus on creating two songs by considering two main ideas: their current experiences at university (e.g., looking at their existing sense of self and wellbeing) and their aspirations for a future self (e.g., looking beyond the institutional boundaries to their own future). These objectives underlay the half-semester program, enabling goal-directed, creative thinking and engagement with an artistic process which concurrently allowed them to explore their sense of self.

**FIGURE 2:**
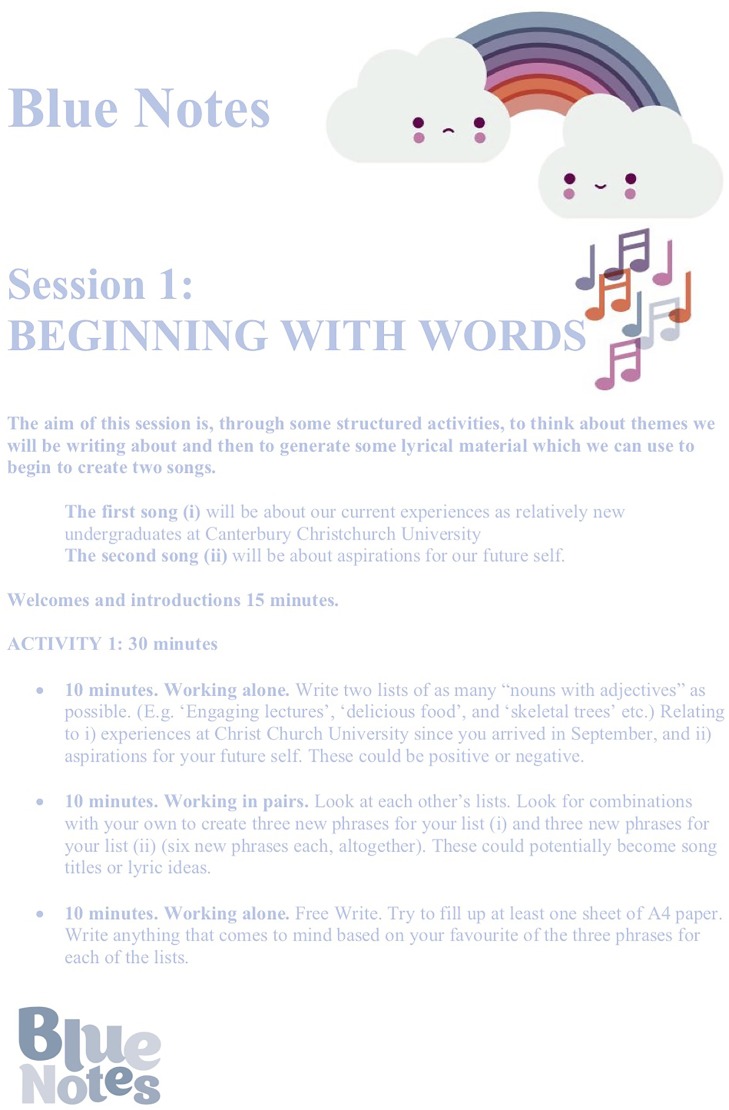
Example of session plan.

Sessions were co-designed and facilitated by a music psychologist and an experienced musician (songwriter, producer, and facilitator) in consultation with a music therapist. A research assistant provided additional workshop support. All of those involved, whether student or facilitator, were engaged in the songwriting workshops as equals. This enabled a working, creative, safe space (Darnley-Smith and Patey, 2006) within the university environment, where students were encouraged to reflect, engage, create and develop, and power dynamics could be kept to a minimum. This followed the therapeutic framework suggestions of Darnley-Smith and Patey (2006), including: the regularity of sessions, placing time boundaries, ensuring the privacy of the space, and using beginnings and endings. Other resources such as “Guidelines for Songwriting” (Aasgaard and Ærø, 2017, p. 650) also shaped the intervention. The facilitators were as non-directive as possible, encouraging metacognition by focussing on ways to express and understand emotions, ideas, sense of self etc. Each session was 2 h and contained three structured activity sessions roughly 20–30 min in duration. Around the longer structured activity sessions were smaller 5–10 min warm up exercises (physical, musical, and lyrical) enabling students to connect with each other in different ways. Space was created in the sessions for students to work alone, in large groups, in small groups and in pairs. Students took homework, and brought new ideas, products, or pieces of pre-existing music with them to subsequent sessions. An example of a session plan is provided in Figure 2.

### Measures

Measures related to mental health and social connection were included in the main study and divided into primary and secondary outcome measures.

#### Primary Outcome Measures

##### Mental health

The primary outcome measure for mental health was The Hospital Anxiety and Depression Scale (HADS) (Zigmond and Snaith, 1983). HADS is used with clinical and sub-clinical populations, and focuses on non-physical symptoms (Stern, 2014). It is one of the NICE recommended diagnostic tools for initial measurement of anxiety and depression (Quality Standard: Depression in Adults, 2011).

The scale comprises two seven item subscales assessing levels of depression (HADS-D) and anxiety (HADS-A). Participants rated how frequently in the previous week they had experienced symptoms, for example: “I can laugh and see the funny side of things” (depression subscale) or “I get sudden feelings of panic” (anxiety subscale). Ratings were taken on a four-point scale (0–3), e.g., 0 = Not at all 3 = Very often indeed. Cronbach’s alpha demonstrates acceptable reliability for HADS-A at α = 0.797 (T1) and α = 0.819 (T2), and for HADS-D at α = 0.904 (T1) and α = 0.670 (T2).

##### Social connection

The primary outcome measure for social connection was the Four-Item Measure of Social Identification (FISI) (Postmes et al., 2013) composed of four items: “I identify with [in-group],” “I feel committed to [in-group],” “I am glad to be [in-group],” “being [in-group] is an important part of how I see myself.” The in-group was “the university” (as opposed to less formal groups such as friendship groups). Items were rated on a seven-point Likert scale (1 = strongly disagree, 7 = strongly agree). Cronbach’s alpha demonstrated acceptable reliability for FISI at α = 0.885 (T1) and α = 0.960 (T2).

#### Secondary Outcome Measures

##### Mental health

A brief version of the Social Phobia Inventory, the mini-SPIN (Connor et al., 2001), was included, which gives an indication of the presence of social anxiety disorder. Participants rated three items on a five-point Likert scale ranging from 0 = not at all to 4 = extremely: “Fear of embarrassment causes me to avoid doing things or speaking to people,” “I avoid activities in which I am the centre of attention,” “being embarrassed or looking stupid are among my worst fears.” This measure explores social phobias in relation to social connections (Stein and Stein, 2008). Its three items discriminate between individuals with generalised social anxiety disorder and controls. α = 0.847 (T1) and α = 0.849 (T2).

##### Social connection

Aspects of social connection were assessed using two secondary measures: The Russel UCLA loneliness scale (Version 3) (UCLA3) (Russell et al., 1978), and the Friendship Item Scale (FIS) (Hawthorne, 2006).

The UCLA3 is a 20-item scale measuring subjective feelings of loneliness and isolation. Specifically, the extent a person perceives they are in relationships and the personal and social levels of those relationships. Participants rate items such as, “I am unhappy doing so many things alone,” “It is difficult for me to make friends,” and “I feel completely alone,” on a four-item scale. O indicated “I often feel this way,” S indicated “I sometimes feel this way,” R indicated “I rarely feel this way,” and N indicated “I never feel this way.” α = 0.874 (T1) and α = 0.923 (T2).

The Friendship Item Scale (FIS) (Hawthorne, 2006) comprised six items referring to social isolation as measured by frequency over the previous 4 weeks (e.g., “I found it easy to get on with other people,” “I felt lonely,” etc.) These were measured on a five-point Likert scale from 1 = Almost always to 5 = Not at all. α = 0.799 (T1) and α = 0.642 (T2). (In the original trial registration, the Satisfaction with Life Scale (SWLS) was one of the measures, however, given this is a pilot study and given the breadth of social wellbeing as a concept, and the emphasis on social connection within this population for this study, the SWLS was replaced by the FIS (Hawthorne, 2006).

#### Additional Measures

The Meaningfulness of Songwriting Scale (MSS) (Baker et al., 2016b) comprised 21 items understanding the value of songwriting to participants, particularly the extent of meaning surrounding the process and the outcomes of songwriting. The MSS measured meaning over 11 domains of meaningfulness, including: enjoyment, discovery/self-reflection, arousal of emotions, creativity, engagement, challenge, understanding context, associations, achievement, personal value, and identity (e.g. “songwriting was a challenging experience,” “songwriting aroused my emotions,” etc.). These were measured on a five-point Likert scale from 1 = Strongly disagree to 5 = Strongly agree. Scores range from 21 to 105, with larger scores indicating stronger meaning being derived from the process and the product. Previous work has illustrated that the scale has adequate measurement properties not only in clinical samples but also with non-music specialist university students (Baker et al., 2016a).

Students were also asked to document the number of times they engaged with current student services, to rate the existing support provided within university, and to make qualitative suggestions about improvements to that provision.

#### Statistical Analysis

Given the size of the sample, data was analysed by taking pre-test and post-test scores, and calculating the difference between these as change scores. Change scores were then subject to a non-parametric Mann–Whitney *U*-test.

Sociodemographics and questionnaire data was also discussed for the sample.

## Results

### Recruitment

Figure 1 illustrates that 44% of the total sample (*n* = 181 first year students) responded to the screening questionnaire survey, these therefore self-selected as stressed, anxious, or depressed. After randomisation to the intervention or wait-list control group, students were invited to take part in the study. 20.5% of the allocated intervention participants agreed to take part (*n* = 7). Gender matched controls were contacted to take part, 100% of the controls asked then agreed to participate in the study. Detailed participant sociodemographics are illustrated in Table 1.

### Pre-intervention Attrition

Although initial interest in the study was substantial, pre-intervention attrition was high once allocation to the intervention had taken place (79% declined to take part). The 27 students who were allocated to the intervention but declined to take part were followed up with an email questionnaire to explore their disengagement. Seventeen responded with reasons, including: “too stressed,” “not enough time to take part,” “too many assignments,” “don’t remember taking part in initial survey,” “it’s not for me.” These responses raise a number of questions pertinent to the pilot study. First understanding of the university environment and its associated pressures and student social norms, particularly ideas around stress and time management, the perceived work-load and significance of assignments. Timing of an intervention within the academic year is also important (avoiding holiday periods). As is the time lag between initial recruitment and study allocation. (This project recruited at the end of semester one, and but the intervention began after reading week in semester two).

### Intervention Attrition and Adherence

Dropout was defined as those students who did not complete the post-intervention questionnaire, regardless of the number of songwriting intervention sessions they attended, or whether they were in the control group. One out of 13 participants did not complete the post-intervention questionnaire. Adherence was good, with attendance at intervention sessions at 90%. All of those asked to take part as a control agreed to do so.

### Intervention Efficacy

Although it is difficult to make any statistical inferences given the sample size, change scores were calculated for the intervention group and control group, and a non-parametric Mann–Whitney *U*-test was conducted. A Mann–Whitney *U*-test was conducted on the pre-post change scores to determine whether there was a difference between songwriters and wait-list control participants on the battery of measures. Results are documented in Table 2.

Significant differences were found for two of the five measures: Levels of depression (HADS-D) in wait-list controls (*Mdn* = 8.5) significantly differed from songwriters (*Mdn* = -1.0) on their pre-post change scores, *U* = 5.00, *z* = -2.085, *p* < 0.041, *r* = 2.66; Social connection levels (FISI) in wait-list controls (*Mdn* = -3.00) significantly differed from songwiters (*Mdn* = -2.50) on their pre-post change scores, *U* = 3.00, *z* = -2.441, *p* < 0.015, *r* = 0.524. T1 T2 and raw scores and change scores for these measures are documented in Table 3.

**Table 2:** Pre-post change scores subject to a Mann–Whitney *U*-test.

	HADS-A change score	HADS-D change score	Friendship change score	Mini-SPIN change score	UCLA change score	FISI change score
Mann–Whitney U	13.00	5.00	8.00	8.00	8.00	3.00
Wilcoxon W	34.00	26.00	29.00	29.00	29.00	24.00
Z	-8.11	-2.085	-1.613	-1.630	-1.604	-2.441
Asymp. Sig. (2-tailed)	0.418	0.037	0.107	0.103	0.109	0.015
Exact Sig. [2^∗^(1-tailed Sig.)]	0.485	0.041	0.132	0.132	0.132	0.015


**Table 3:** Time 1 and Time 2 data for HADS-D and FISI measures.

Participant	T1 HADS-D	T2 HADS-D	Change score	T1-FISI	T2-FISI	Change score
C1	6	15	9	22	19	-3
C2	0	13	13	24	23	-1
C3	5	13	8	25	22	-3
C4	4	15	11	16	19	3
C5	18	9	-9	22	19	-3
C6	4	11	7	10	6	-4
I1	4	4	0	21	25	4
I2	8	7	-1	21	26	5
I3	7	5	2	25	26	1
I4	4	3	-1	24	28	4
I5	16	6	-10	26	27	1
I6	11	5	-6	24	25	1


*C1, Control participant 1; I1, Intervention participant 1; The higher the HADS score the higher levels of depression. The higher the FISI score the greater the social connection to the university.*

Anxiety levels (HADS-A) in wait-list controls (*Mdn* = 0.00) did not significantly differ from songwriters (*Mdn* = -2.5) on their pre-post change scores, *U* = 13.00, *z* = -0.811, *ns, r* = 3.229. The measure of Friendship in wait-list controls (*Mdn* = 0.00) did not significantly different from songwriters (*Mdn* = 6.0) on their pre-post change scores, *U* = 8.00, *z* = -1.613, *ns, r* = 2.162. Measure of social anxiety (Mini_Spin) in wait-list controls (*Mdn* = -1.00) was not significantly different from songwriters (*Mdn* = 1.00) on their pre-post change scores, *U* = 8.00, *z* = -1.630, *ns, r* = 1.691. Loneliness levels (UCLA) in wait-list controls (*Mdn* = 0.50) did not differ significantly from songwriters (*Mdn* = -7.0) on their pre-post change scores, *U* = 8.00, *z* = -1.604, *ns, r* = 1.228.

**Table 4:** Survey questions documenting self-report thoughts on elements of the project.

83% said the intervention had improved their transition to university life and made them feel more settled
67% stated the intervention had improved their mental wellbeing
67% experienced an increase in confidence during the intervention
100% agreed that they now felt like an integral part of the university
100% reported that they found the sessions beneficial, enjoyable and wanted to continue.


Participants also responded to survey questions asking them to qualitatively self-report their thoughts on aspects of the intervention, presented in Table 4.

### Meaningfulness of Songwriting

Meaningfulness of Songwriting Scale (MSS) measures the extent of meaning found within the songwriting process and outcomes. Meaning scores fall between 21 and 105 points, with higher scores indicating greater meaning. This sample ranged between 76 and 104 points, with a mean score of 85.5, and SD of 9.874. 66% of the scores fell in the top quarter of the range, indicating a level of inherent meaning in the process and product of songwriting. Songwriting is therefore a therapeutic intervention that holds substantial meaning for this population.

## Discussion

### Support for Songwriting as a Wellbeing Intervention in a Student Population

Songwriting shows promise as a therapy for use with a student population with pre-existing mental health difficulties. This pilot study illustrated both the prevalence of perceived stress, depression and anxiety in a student population, the isolation of students within this environment with mental health difficulties, and the potential benefits of arts interventions held in non-clinical, non-community, environments.

The intervention may effect levels of depression (HADS-D) and social connection (FISI). Closer analysis of the T1 and T2 scores (Table 3) shows that although there was a trend toward a reduction in depression scores for the songwriters, the greater change came from the heightened increase in the depression scores for the control group. It may be therefore that songwriting is acting as a preventative mechanism for these students in this environment, given that there was a clear increase in depression scores for the control group. However, with such a small sample size these findings need to be interpreted with caution and the effects of this type of group intervention need to be studied in more detail.

When considering the scores for social connection to the university – wider social identity – the songwriters’ scores have strengthened: means were 23.5 (T1) to 26.1 (T2), compared to controls whose means were 19.8 (T1) to 18 (T2). An argument can be made that a songwriting intervention helps to bolster social identity, the connection with the university, and may act as mechanism of support. When faced with the various stresses of university, this connection helps songwriters to cope, rather than the pressures exacerbating mental health distress which was the case for wait-list controls. This is partially consistent with previous research suggesting that songwriting can positively effect depression in clinical populations (Erkkilä et al., 2011) and have a positive impact on student populations (Baker et al., 2016a, 2017). From a social identity approach these findings fit with ideas in previous work exploring wellbeing and groups. Haslam et al. (2014) suggested that when conducting song-based reminiscence interventions in care homes, that social identification with the wider care home was implicit in the efficacy of the intervention in producing positive health outcomes. This songwriting pilot fits with recent social identity work, suggesting that life transitions (particularly that of being a student) can be supported through activation of and belonging to multiple social groups. Ng et al. (2018, p. 173) suggest that the SIMIC model could be used to understand wellbeing and the student experience; “Group memberships generally make a positive contribution to individuals’ wellbeing, especially in the context of adjusting to life transitions…evidence suggests that having multiple identities is especially important in periods of vulnerability.” They further suggest that “joining new groups can be a powerful way of staving off the negative effects of social isolation associated with a lack, or loss of social identity and thereby reducing the risk of developing depression, anxiety, and stress as a result.”

The battery of measures was selected to cover a spread of clinical and subjective wellbeing measures which could be affected by songwriting. In selecting this population and specific measures, this paper explore two of Baker’s objectives: Understanding the aspects of wellbeing that are positively impacted by songwriting, and expanding the populations that songwriting is used with from a quasi-therapeutic perspective. Qualitative reports suggest that the battery could be adapted with additional or replacement measures looking at confidence, social connection, and wellbeing.

### Unsupported Hypotheses

Given the high self-reported levels of anxiety, and specific growth in perfectionism and associated anxiety in the student body over time (Curran and Hill, 2017) a strong reduction in anxiety between wait-list control and songwriting intervention groups would have been expected. However, neither HADS-A, nor associated social phobia scales (mini-SPIN) saw any significant changes between the two groups.

Measures of friendship (FIS) and loneliness (UCLA3) did not show any significant changes between the two groups. Perhaps this is a little surprising given the significant differences in social connection between the control and intervention groups. It is probable that social connection, friendship, and loneliness are distinct, unrelated concepts – with social connection operating at a group social identity level, whereas friendship and loneliness may operate on a more individualistic personal identity level, which was left unaffected by this group intervention. However, again these hypotheses must be cautiously interpreted due to the sample size of the pilot trial, and it is recommended that a future RCT would adapt and develop the battery.

### Exploring the Efficacy of the Trial

Feedback relating to attrition rates was centred on student perceptions of university as a space that was stressful and assessment focussed, rather than on the unsuitability of the intervention, or fear of a novel or unusual intervention. This may raise important questions about the changing university environment, students’ education expectations, and widening a student’s horizons to non-curriculum-based activities. This study supports the idea of finding room for support and enabling space for engagement beyond the curriculum and outside of the stereotypical student societies and associated social norms.

The study sample was self-selecting and were all female, which may have been unavoidable, given the pool the project drew from (both psychology and music being female-dominated courses). This may have changed the dynamic of the sessions or relationships that were formed during the project.

Unfortunately, due to financial year end, it was impossible to offer the wait-list control the intervention, or to invite the songwriting cohort to continue the study in the next academic year. Ideally a cross-over design, with participants serving as their own controls, would have been used. This would have reduced variability and minimised sample size to achieve adequate power, whilst counterbalancing any order effects. Efficacy could also have been examined without denying treatment to either group. Given the complex nature of music therapy interventions, some suggest that RCTs are limited within this type of work and practical trials, preference trials or propensity score methodology could be used instead (Robb and Burns, 2017).

### Implications of the Research and Applications

A therapeutic songwriting intervention is novel within higher education settings, but looking to other recent studies involving non-clinical participants (Baker et al., 2018), we can begin to speculate as to some of the underlying processes which may occur through this type of intervention with a student population. Baker et al. (2018) documents a six-session group songwriting program for caregivers of people living with dementia. The songwriting process,

“Allowed participants to share their entire caregiver journey with others, differentiating the intervention from standard carer support groups. Participants described group songwriting as enabling them to find connections with other caregivers, create a group identity, and gain insight into their carer journey.”

Similarly, by bringing together a group of students with pre-existing mental health difficulties, we may start to enable an otherwise disparate collective of students to find connections with each other, create a new group identity – whether as songwriters, students who share a common experience, or as students who may gain insight into their student journey. These ideas are explored in the qualitative follow up to this paper.

We do know that this project held intrinsic value for the participants, given their participation and rate of attendance. Also that there was some significance and intrinsic value of the practice and art of songwriting; the participants held relatively high scores on the MSS. However, could it still be argued that students might have taken part in any group activity and received the same benefits, which poses the question: is change a by-product of social connection (being part of a social group, and working on new social identities), or is it the act of songwriting itself that produces positive change? Given the combination of depression (HADS-D), social connection (FISI), and meaningfulness in songwriting (MSS) scores, this work suggests that songwriting in this context and through this mode of delivery, may provide a meaningful base for identification, change, and personal development through the act of participating in a shared creative experience. This idea is certainly supported in the musicology of popular music studies literature, which suggests that young people engage with music to develop their identities through social contact in a popular music environment (e.g., gigs, fanzines etc.) (Frith, 1998). They create and engage activities which enable them to feel part of a social group, and music can become a “badge of identity” (North and Hargreaves, 1999). It may also be worth exploring this type of intervention through a social psychology framework; Praharso et al. (2017) suggest a SIMIC model of health can be used to understand the loss of social identity and subsequent decrease in wellbeing as a result of a life stressful event (such as starting university). Koni et al. (2019) develop this idea within an applied setting, by exploring the impact of providing adolescents with a new group (i.e., a new social identity) on psychological resilience. Their work illustrated that creating a new social identity through an active group intervention worked best more vulnerable adolescents (similar to those who took part in this study).

Student retention is recognised as a significant institutional challenge, with HESA data indicating a rise from 2011 to 2016 (most recent data) within undergraduates who are under 21 (Non-continuation Summary, 2018, March 8). A recent “What Works” paper (Thomas et al., 2017) provided guidance concerning interventions around retention and success: Effective interventions needed to be both proactive and developmental, with activities “proactively seeking to engage students”; well-timed, with early engagement to promote retention, alongside a variety of media used to convey information; collaborative, with activities encouraging collaboration and engagement between students and staff; monitored, with the extent and quality of students’ engagement being documented and followed up. A songwriting intervention, although a creative and therapeutic tool, as opposed to being strictly curricula or retention based program, naturally fits many of Thomas et al.’s (2017) ideas for effective retention programs.

Another perspective to take on songwriting interventions, is to examine existing support services within university environments. More often than not these services are often overstretched, and so there is a tendency within institutions to look toward technology or “E-solutions” for health and wellbeing difficulties in this population (Prosser et al., 2018). In contrast, however, this project suggests that there is clear and special value in face-to-face, relational, group work with this particularly vulnerable population. The project’s findings also indicate that wellbeing interventions could go beyond the standard provision of university services, through the use of novel, creative approaches, with arts therapists and practitioners.

## Data Availability

The raw data supporting the conclusions of this manuscript will be made available by the authors, without undue reservation, to any qualified researcher.

## Ethics Statement

This study was carried out in accordance with the recommendations of requirements for proportionate ethical review as set out in Canterbury Christ Church’s University’s Research Ethics and Governance Procedures. The protocol was approved by the Research Governance Committee. The British Psychological Society’s ethical guidelines were applied throughout this study. All subjects gave written informed consent in accordance with the British Psychological Society guidelines.

## Author Contributions

KG conceived of the trial, conducted the literature review, consulted with various experts, designed the intervention, participated in the songwriting sessions, designed data collection tools, monitored data collection for the whole trial, wrote the statistical analysis plan, conducted the statistical analysis, and wrote the initial and final drafts of the research manuscript. NC designed the intervention, facilitated the weekly songwriting sessions, recorded, and produced the artistic products and previewed the final versions of the research manuscript. VH supported recruitment and the writing and editing of the final research manuscript.

## Conflict of Interest Statement

The authors declare that the research was conducted in the absence of any commercial or financial relationships that could be construed as a potential conflict of interest.

## Footnotes

1 www.qualtrics.com

2 www.random.org

## References

[B1] Aasgaard T. Ærø S. C. B. (2017). “‘Songwriting Techniques in Music Therapy Practice’,” in *The Oxford Handbook of Music Therapy*, 1st edn, ed. Jane E. (Oxford: Oxford University Press).

[B2] Baker F. A. (2015). *Therapeutic Songwriting: Developments in Theory, Methods, and Practice*, 1st edn. Basingstoke: Palgrave MacMillan 10.1057/9781137499233

[B3] Baker F. A. MacDonald R. A. R. (2013). Flow, identity, achievement, satisfaction and ownership during therapeutic songwriting experiences with university students and retirees. *Music. Sci.* 17 131–146. 10.1177/1029864913476287

[B4] Baker F. A. Jeanneret N. Clarkson A. (2017). ‘Contextual factors and wellbeing outcomes: ethnographic analysis of an artist-led group songwriting program with young people’. *Psychol. Music* 46:030573561770952 10.1177/0305735617709520

[B5] Baker F. A. MacDonald R. A. R. Pollard M. C. (2016a). Reliability and validity of the meaningfulness of songwriting scale with university students taking a popular songwriting class. *Arts Health* 10 1–12. 10.1080/17533015.2016.1236281

[B6] Baker F. A. Silverman M. J. MacDonald R. A. R. (2016b). ‘Reliability and validity of the meaningfulness of songwriting scale (MSS) with adults on acute psychiatric and detoxification units’. *J. Music Ther.* 53 55–74. 10.1093/jmt/thv020 26673954

[B7] Baker F. A. Stretton-Smith P. Clark I. N. Tamplin J. Lee Y. C. (2018). ’A group therapeutic songwriting intervention for family caregivers of people living with dementia: a feasibility study with thematic analysis’. *Front. Med.* 5:151. 10.3389/fmed.2018.00151 29872659 PMC5972290

[B8] Baker F. A. Wigram T. (2005). *Songwriting: Methods, Techniques and Clinical Applicaitons for Music Therapy Clinicians, Educators and Students.* Philadelphia: Jessica Kingsley Publishers.

[B9] Boothby D. M. Robbins S. J. (2011). The Effects of Music listening and art production on negative mood: a randomized, controlled trial. *Arts Psychother.* 38 204–208. 10.1016/j.aip.2011.06.002

[B10] Choi A.-N. Lee M. S. Lim H.-J. (2008). ‘Effects of group music intervention on depression, anxiety, and relationships in psychiatric patients: a pilot study’. *J. Alter. Compl. Med.* 14 567–570. 10.1089/acm.2008.0006 18564958

[B11] Clift S. Hancox G. Morrison I. Shipton M. Page S. Skingley A. (2016). “‘Group Singing as a Public Health Resource,’” in *Oxford Textbook of Creative Arts, Health, and Wellbeing. International Perspectives on Practice, Policy and Research*, 1st ed, eds Stephen C. Paul C. (Oxford: Oxford University Press), 251–259.

[B12] Cohen M. L. Miller P. (2017). “Dear Younger Me”: Writing, Songwriting, and Choral Singing While Incarcerated as a Means to Build Identities and Bridge Communities,” in *Evidence and Impact in Theatre, Music and Art*, eds Matthew R. Nick R. (London: Bloomsbury).

[B13] Connor K. M. Kobak K. A. Churchill L. E. Katzelnick D. Davidson J. R. T. (2001). ‘Mini-SPIN: a brief screening assessment for generalized social anxiety disorder’. *Depress. Anxiety* 14 137–140. 10.1002/da.1055 11668666

[B14] Coulton S. Clift S. Skingley A. Rodriguez J. (2015). ‘Effectiveness and cost-effectiveness of community singing on mental health-related quality of life of older people: randomised controlled trial’. *Br. J. Psychiatry* 207 250–255. 10.1192/bjp.bp.113.129908 26089304

[B15] Craig P. Dieppe P. Macintyre S. Michie S. Nazareth I. Petticrew M. (2019). *‘Developing and Evaluating Complex Interventions: New Guidance’.* Available at www.mrc.ac.uk/complexinterventionsguidance

[B16] Craig P. Dieppe P. Macintyre S. Mitchie S. Nazareth I. Petticrew M. (2008). ‘Developing and evaluating complex interventions: the new medical research council guidance’. *BMJ* 337:a1655. 10.1136/bmj.a1655 18824488 PMC2769032

[B17] Cromby J. Harper D. Reavey P. (2013). *Psychology, Mental Health and Distress.* Basingstoke: Palgrave MacMillan 10.1007/978-1-137-29589-7

[B18] Cruwys T. Haslam S. A. Dingle G. A. Haslam C. Jetten J. (2014). ‘Depression and social identity: an integrative review’. *Pers. Soc. Psychol. Rev.* 18 215–238. 10.1177/1088868314523839 24727974

[B19] Curran T. Hill A. P. (2017). Perfectionism is increasing over time: a meta-analysis of birth cohort differences from 1989 to 2016. *Psychol. Bull.* [Epub ahead of print]. 10.1037/bul0000138 29283599

[B20] Darnley-Smith R. Patey H. M. (2006). *Music Therapy.* London: Sage Publication Ltd.

[B21] Davies E. (2005). “‘You Ask Me Why i’m Singing: Song-Creating with Children at a Child and Family Psychiatric Unit’,” in *Songwriting Methods, Techniques and Clinical Applications for Music Therapy Clinicians, Educators and Students*, eds Felicity A. B. Tony W. (Philadelphia: Jessica Kingsley Publishers).

[B22] Daykin N. Mansfield L. Meads C. Julier G. Tomlinson A. Payne A. (2018). What works for wellbeing? A systematic review of wellbeing outcomes for music and singing in adults. *Perspect. Public Health* 138 39–46. 10.1177/1757913917740391 29130840 PMC5753835

[B23] Eldridge S. M. Chan C. L. Campbell M. J. Bond C. M. Hopewell S. Thabane L. (2016a). CONSORT 2010 statement: extension to randomised pilot and feasibility trials. *BMC* 355:i5239. 10.1136/bmj.i5239 27777223 PMC5076380

[B24] Eldridge S. M. Lancaster G. A. Campbell M. J. Thabane L. Hopewell S. Coleman C. L. (2016b). Defining feasibility and pilot studies in preparation for randomised controlled trials: development of a conceptual framework. *PLoS One* 11:e0150205. 10.1371/journal.pone.0150205 26978655 PMC4792418

[B25] Erkkilä J. Punkanen M. Fachner J. Ala-Ruona E. Pöntiö I. Tervaniemi M. (2011). ‘Individual music therapy for depression: randomised controlled trial’. *Br. J. Psychiatry* 199 132–139. 10.1192/bjp.bp.110.085431 21474494

[B26] Frith S. (1998). *Performing Rites: On The Value of Popular Music.* Massachusetts: Harvard University Press.

[B27] Hanser S. B. Bauer-Wu S. Kubicek L. Healey M. Manola J. Hernandez M. (2006). Effects of a music therapy intervention on quality of life and distress in women with metastatic breast cancer. *J. Soc. Int. Oncol.* 4 116–124. 10.2310/7200.2006.014 19442346

[B28] Haslam C. Haslam S. A. Ysseldyk R. McCloskey L. G. Pfisterer K. Brown S. G. (2014). ‘Social identification moderates cognitive health and well-being following story- and song-based reminiscence.’. *Aging Ment. Health* 18 425–435. 10.1080/13607863.2013.845871 24131035

[B29] Hawthorne G. (2006). ‘Measuring social isolatin in older adults: development and initial validation of the friendship scale’. *Soc. Indic. Res.* 77 521–548. 10.1007/s11205-005-7746-y

[B30] Iyer A. Jetten J. Tsivrikos D. Postmes T. Haslam S. A. (2009). ‘The more (and the more compatible) the merrier: multiple group memberships and identity compatibility as predictors of adjustment after life transitions’. *Br. J. Soc. Psychol.* 48 707–733. 10.1348/014466608X397628 19200408

[B31] Jetten J. Haslam C. Haslam S. A. Branscombe N. R. (2009). *‘The Social Cure’. Scientific American Mind.* New York, NY: Psychology Press.

[B32] Kerr H. (2013). *‘Mental Distress Survey Overview’. NUS Services Limited.* https://www.nus.org.uk/Global/Campaigns/20130517%20Mental%20Distress%20Survey%20%20Overview.pdf

[B33] Klein C. M. Silverman M. J. (2012). With love from me to me: using songwriting to teach coping skills to caregivers of those with alzheimer’s and other dementias. *J. Creativ. Ment. Health* 7 153–164. 10.1080/15401383.2012.685010

[B34] Koni E. Moradi S. Arahanga-Doyle H. Neha T. Hayhurst J. G. Boyes M. (2019). Promoting resilience in adolescents: a new social identity benefits those who need it most. *PLoS One* 14:e0210521. 10.1371/journal.pone.0210521 30629716 PMC6328232

[B35] Mcnamara R. J. Epsley C. Coren E. Mckeough Z. J. (2017). Singing for adults with chronic obstructive pulmonary disease (COPD). *Cochrane Database Syst. Rev.* 12:CD012296. 10.1002/14651858.CD012296.pub2 29253921 PMC5835013

[B36] Ng N. W. K. Haslam S. A. Haslam C. Cruwys T. (2018). How can you make friends if you don’t know who you are?” a qualitative examination of international students’ experience informed by the social identity model of identity change. *J. Commun. Appl. Soc. Psychol.* 28 169–187. 10.1002/casp.2349

[B37] Non-continuation summary. (2018). *Non-Continuation Summary: UK Performance Indicators 2016/17.* Available at https://www.hesa.ac.uk/news/08-03-2018/non-continuation-summary

[B38] North A. C. Hargreaves D. J. (1999). ‘Music and adolescent identity’. *Music Educ. Res.* 1 75–92. 10.1080/1461380990010107

[B39] O’Brien E. (2014). *The Effect and Experience of Therapeutic Songwriting on Adult Cancer Patients’ Quality of Life, Mood, Distress Levels and Satisfaction With Hospital Stay.* Ph.D. thesis Melbourne, VIC: University of Melbourne.

[B40] O’Cathain A. Goode J. Drabble S. J. Thomas K. J. Rudolph A. Hewison J. (2014). ‘Getting added value from using qualitative research with randomized controlled trials: a qualitative interview study’. *Trials* 15:215. 10.1186/1745-6215-15-215 24913438 PMC4059032

[B41] Postmes T. Haslam S. A. Jans L. (2013). ‘A single-item measure of social identification: reliability. validity, and utility’. *Br. J. Soc. Psychol.* 52 597–617. 10.1111/bjso.12006 23121468

[B42] Praharso N. F. Morgan J. Cruwys T. (2017). ’Stressful life transitions and wellbeing: a comparison of the stress buffering hypothesis and the social identity model of identity change.’. *Psychiatry Res.* 247 265–275. 10.1016/j.psychres.2016.11.039 27936438

[B43] Prosser T. Gee K. A. Jones F. (2018). ‘A meta-analysis of effectiveness of e-interventions to reduce alcohol consumption in college and university students’. *J. Am. College Health* 16 1–25. 10.1080/07448481.2018.1440579 29452058

[B44] Quality Standard: Depression in Adults. (2011). *National Institute for Health and Care Excellence.* Available at https://remote-lib.ui.ac.id:2194/#!/content/medical_topic/21-s2.0--1011026

[B45] Reason M. Rowe N. (2017). *Evidence and Impact in Theatre, Music and Art*, eds Matthew R. Nick R. London Bloomsbury.

[B46] Robb S. L. Burns D. S. (2017). “‘Randomised Controlled Trials in Music Therapy’,” in *The Oxford Handbook of Music Therapy*, ed. Jane E. (Oxford: Oxford University Press).

[B47] Russell D. Peplau L. A. Ferguson M. L. (1978). ‘Developing a measure of loneliness’. *J. Pers. Assess.* 42 290–294. 10.1207/s15327752jpa6601 660402

[B48] Shwu Ming W. U. (2002). ‘Effects of music therapy on anxiety, depression and self-esteem of undergraduates. *Psychologia* 45 104–114. 10.2117/psysoc.2002.104

[B49] Silverman M. J. (2011). ‘The effect of songwriting on knowledge of coping skills and working alliance in psychiatric patients: a randomized clinical effectiveness study’. *J. Music Ther.* 48 103–122. 10.1093/jmt/48.1.103 21866716

[B50] Silverman M. J. (2013). ‘Effects of group songwriting on depression and quality of life in acute psychiatric inpatients: a randomised three group effectiveness study’. *Nord. J. Music Ther.* 22 131–148. 10.1080/08098131.2012.709268

[B51] Silverman M. J. Baker F. A. MacDonald R. A. R. (2016). ‘Flow and meaningfulness as predictors of therapeutic outcome within songwriting interventions’. *Psychol. Music* 44 1331–1345. 10.1177/0305735615627505

[B52] Sneyers E. De Witte K. (2018). ‘Interventions in higher education and their effect on student success: a meta-analysis’. *Educ. Rev.* 70 208–228. 10.1080/00131911.2017.1300874

[B53] Southgate E. (2019). Freshers declaring mental illness up 73% in 4 years. *The Times*, 22nd February, 4.

[B54] Stegemöller E. L. Radig H. Hibbing P. Wingate J. Sapienza C. (2017). Effects of singing on voice, respiratory control and quality of life in persons with parkinson’s disease. *Disabil. Rehabil.* 39 594–600. 10.3109/09638288.2016.1152610 26987751

[B55] Stein M B. Stein D. J. (2008). Social anxiety disorder. *Lancet* 371 1115–1125. 10.1016/S0140-6736(08)60488-2 18374843

[B56] Stern A. F. (2014). Questionnaire review: the hospital anxiety and depression scale. *Occup. Med.* 64 393–394. 10.1093/occmed/kqu024 25005549

[B57] Tajfel H. (1978). *Differentiation Between Groups: Studies in Social Psychology of Intergroup Relations.* London: Academic Press.

[B58] Thabane L. Hopewell S. Bond C. M. Coleman C. L. Campbell M. J. Eldridge S. M. (2016). Methods and processes for development of a consort extension for reporting pilot randomized controlled trials. *Pilot Feasibil. Stud.* 2:25. 10.1186/s40814-016-0065-z 27965844 PMC5153862

[B59] Thomas L. Hill M. O’Mahony J. Yorke M. (2017). *Supporting Student Success: Strategies For Institutional Change. What Works? Student Retention & Success Programme. Report for What Works?* Available at https://www.phf.org.uk/wp-content/uploads/2017/04/Full-report-Final.pdf

[B60] Thorley C. (2017). *Not By Degrees: Improving Student Mental Health in the UK’s Universities.* Available at http://www.ippr.org/research/publications/not-by-degrees

[B61] Valentine E. Evans C. (2001). ‘The effects of solo singing, choral singing and swimming on mood and physiological indices. *Br. J. Med. Psychol.* 74 115–120. 10.1348/000711201160849 11314898

[B62] van der Steen J. T. van Soest-Poortvliet M. C. van der Wouden J. C. Bruinsma M. S. Scholten R. J. Vink A. C. (2017). ‘Music-based therapeutic interventions for people with dementia’. *Cochrane Database Syst. Rev.* 5:CD003477. 10.1002/14651858.CD003477.pub3 28462986 PMC6481517

[B63] von Lob G. Camic P. Clift S. (2010). The use of singing in a group as a response to adverse life events. *Int. J. Ment. Health Promo.* 12 45–53. 10.2739/kurumemedj.MS652004 30487379

[B64] Zigmond A. S. Snaith R. P. (1983). ‘The hospital anxiety and depression scale’. *Acta Psychiatr. Scand.* 67 361–370. 10.1111/j.1600-0447.1983.tb09716.x 6880820

